# Concern about Environmental Pollution: How Much Difference Do Race and Ethnicity Make? A New Jersey Case Study

**DOI:** 10.1289/ehp.7611

**Published:** 2004-12-23

**Authors:** Michael R. Greenberg

**Affiliations:** Edward J. Bloustein School of Planning and Public Policy, Rutgers University, New Brunswick, New Jersey, USA

**Keywords:** concern, environment, ethnicity, public perspectives, race

## Abstract

A survey conducted among 1,513 residents of New Jersey during March–May 2004 showed that non-Hispanic black, non-Hispanic white, and English-speaking Hispanic Americans were significantly more concerned about environmental pollution problems than were Asian Americans and Spanish-language Hispanic Americans. For example, an average of > 40% of the first three groups was very concerned about New Jersey’s environmental problems, compared with 15% of the last two populations. There were also racial/ethnic differences among these groups in their desire for government action to protect the environment and in their personal support of the environmental movement. Regression analyses suggest that the 1970s and 1980s model of core support for environmental protection from white, female, young, educated, and politically liberal people has largely, but not completely, continued among non-Hispanic white, non-Hispanic black, and English-language Hispanic populations. But these demographic pointers do not hold for Asian and Spanish-language Hispanic Americans, except indicating more support among the more formally educated. The last two groups are the two fastest-growing subpopulations in the United States, and although acculturation may slowly increase their concern about environmental pollution, it is more prudent for proponents of environmental protection not to wait and instead to try to better understand the environmental perceptions of these groups.

This commentary is about the confluence of racial/ethnic identity, other demographic characteristics, and public concern about the environment. Reports show that environmental concern is cyclical, responding to competing issues, most notably a struggling economy and war (Carroll 2002; Crabtree 2003; Gallup Organization 2003; Greenberg 2004; Saad 2003, 2004). Yet we should not focus so completely on cyclical fluctuations that we ignore a potential decline in concern about environment protection in the United States related to the changing racial/ethnic composition of our population. Recent studies show that major differences between white and non-white Americans in concern about the environment, first measured in the 1970s, have declined (Crabtree 2003; Mitchell 1978; Saad 2003). But I would feel more confident about these observations if they were based on multiple studies with sample sizes of 250–400 in each racial/ethnic group instead of a typical random sample of 800–1,600 Americans where the sample sizes of the minority groups are 50–100 for each nonwhite group. Because the non-European population has grown so rapidly and will continue to do so, and because the literature shows that environmental concern is culturally dependent (Bronfman and Cifuentes 2003; Chuk-ling Lai and Tao 2003; Jianguang 1994; Karpowicz-Lazreg and Mullet 1993), I believe that we must not take support for environmental protection from rapidly increasing Asian American and Hispanic American populations for granted. Hence, I conducted a study to collect relatively equal numbers of non-Hispanic whites, non-Hispanic blacks, Asian, and Hispanic Americans in New Jersey to answer two questions: How comparatively concerned are non-Hispanic whites, non-Hispanic blacks, Asian Americans, and Hispanic Americans about environmental protection? And how much variation in concern about environmental protection is associated with racial and ethnic identity compared with income, education, age, sex, language, and perceived local environmental quality?

## Materials and Methods

For purposes of this article, the population that I call “white” are non-Hispanic white or European American, and the group I label “black” are non-Hispanic black or African American. All persons who self-identified as Hispanic or Latino were labeled “Hispanic,” even if they also said that they were white or black. This practice avoids double-counting of respondents.

This commentary is based on a telephone sample survey of New Jersey residents by three university-based organizations. Households were contacted through random-digit dialing, which gives every residential telephone number in New Jersey a mathematically equal chance of being called for an interview. The, survey of 1,513 adults, ≥ 18 years of age, was begun before Earth Day in March 2004 and concluded in May 2004. The goal was to obtain at least 350 non-Hispanic white, 350 non-Hispanic black, 350 Hispanic, and 250 Asian responses. The lower target set for Asian-American respondents reflects the reality that the chances of contacting an Asian respondent via random-digit dialing is less in New Jersey than for other groups because the population constitutes only about 6% of the state’s population. Given the demographics of New Jersey, interviewers were available to conduct interviews in Spanish, Korean, and Chinese. Surveyors phoned, and the first two questions they asked screened for race/ethnicity and language preference. When a respondent expressed a desire to be interviewed in a language other than English, interviewers shifted to that call.

Like every survey method, random-digit dialing has limitations. People who do not own phones, who use mobile phones, or who rarely are at home will be underrepresented in the survey—typically poor and young. The standard way of reducing sampling bias is to weight the sample so that it matches the population as a whole. For example, if 18- to 24-year-olds are 12% of the population but represent only 8% of the sample, then each 18- to 24-year-old in the survey is weighted to count for 1.5 people (12/8). Weighting, however, does not fully solve the sampling bias problem because the 18- to 24-year-olds in the survey may be different from 18- to 24-year-olds who were missed by the sample.

The survey contained 59 questions, of which 41 were used. Twenty-one of the questions were used to measure five elements of environmental concern: *a*) environmental problems, *b*) call for government action to protect the environment, *c*) personal support for environmental protection, *d*) trust of government and science to protect the environment, and *e*) concern about other societal problems. Six of the 21 questions asked how serious were environmental problems related to the disposal of toxic wastes, water pollution, disposal of solid waste and garbage, pollution of beaches, loss of open space, and air pollution. Notably, all but the open space question were about environmental pollution. The requested responses were “very serious,” “somewhat serious,” or “not too serious.” The seventh question asked for an overall judgment about the severity of environmental problems in New Jersey. The options were “very serious,” “somewhat serious,” and “not too serious.”

The next set of four questions asked about the desire for government action. Three asked for judgments about the federal, state, and their local government’s efforts to protect the environment (“too much,” “too little,” “about the right amount”). A fourth asked whether respondents wanted to maintain current antipollution laws even if it meant fewer jobs. The last question has been a standard in surveys for decades, so it was included. However, it poses a dichotomy that, if not false (choice between economy and environment), certainly poses a choice that predisposes more blue-collar workers than white-collar ones to choose weakening the environmental laws. The other three questions have no obvious group selection bias.

The third element was personal support for environmental protection. One question asked about personal involvement, by asking respondents to indicate the extent to which they were supporters or opponents of environmental protection. Responses were “active supporter,” “supporter, but not active,” “opponent, but not active,” and “active opponent.”

The fourth set of concern questions was about trust. One asked if they trusted the New Jersey State Department of Environmental Protection as a source of information about environmental problems (four-point scale from “strongly agree” to “strongly disagree”). A second was a more global question, which asked about overall trust of science to settle differences of opinion about environmental risks. The question asked respondents the extent to which they felt that they have control over risks to their health. My assumption was that those who felt that they had less control would be less trusting of state officials and science. This assumption rests on evidence that trust in institutions increases when the economy is healthy, crime is down, and people feel more comfortable and in control (Pew Research Center 1998).

Six questions targeted other issues to be concerned about in New Jersey, which is the most densely populated and affluent state in the United States. They asked for an assessment of the following: rats, vermin and uncontrolled animals, drug-related crime, obesity, terrorism, traffic congestion, and urban redevelopment. I assumed that those who were concerned about these problems would also be concerned about the environment. Specifically, people who are politically liberal, I assumed, would tend to be more concerned about crime, traffic congestion, and all of these problems than those who were politically conservative. Yet there should be geographic variation in responses. Those who were residents of cities and industrial suburbs would be more likely to be concerned about crime and rats, whereas their counterparts in the suburbs would be, I expected, more concerned about traffic congestion.

Fourteen of the remaining 20 questions were used to create demographic and geographic correlates of public perception: race/ethnicity, age, sex, educational achievement, income, and political identification (liberal, conservative, neither). Three of the 14 questions measured geographic attributes. One question asked respondents to rate New Jersey as a place to live, and a second, their own neighborhood as a place to live. The choices were “excellent,” “good,” “fair,” and “poor” quality. The last question asked their length of residence in New Jersey. The assumption was that environmental concern would be higher among those who were distressed by the surrounding environment and had lived in the area for many years and not become inured to what they perceived as a poor or fair quality environment.

The last six questions assumed that source of information influences perception (Kasperson et al. 1988; Krimsky and Plough 1987). We asked respondents to tell us which sources of information they relied on for “accurate” and “reliable” information about the environment. The choices included the Internet, family and friends, community and civic organizations, television, radio, and newspapers. The underlying hypothesis was that nonmainstream Americans would disproportionately trust family and friends, and civic organizations (Krimsky and Plough 1987). In the context of environmental protection, I define “mainstream” Americans as English-speaking, long-term residents of the United States who had been exposed to Earth Day and post-Earth Day discussions in the mass media about the importance of stopping pollution. Nonmainstream Americans, in this context, are relatively recent immigrants.

With regard to methods, I used a variety of simple and multivariate statistical methods to answer the two research questions. To answer the first question (differences by major racial/ethnic group), I used simple one-way analyses of variance (ANOVA) to compare measures of concern across the four racial/ethnic groups.

With regard to the second research question (demographic and geographic correlates of concern), preliminary analyses were conducted, and the results of these are briefly reviewed. Cronbach’s α was used to determine if we could build a single “environmental pollution concern” scale to correlate with race/ethnicity, age, and other demographic and environmental correlates. A Cronbach α of 0.729 was computed from the 21 environmental concern variables, where a score of ≥ 0.7 is considered evidence of a “good” scale and ≥ 0.8 is considered evidence of an “excellent” single scale. Hence, I could calculate a single concern scale to answer question 2 rather than conduct five different sets of analyses.

I used ordinary least-squares regression models to answer the second question about the demographic correlates of variation in environmental concern. Specifically, three regression models were created. One assumes that we know only the racial/ethnic composition of respondents. The second assumes that we do not know the racial/ethnic composition but that we do know age, education, income, and other demographic and geographic characteristics. These first two models are naive models insofar as they allow no interaction between race/ethnicity and other demographic characteristics. The third and the most realistic model includes interactions among all the statistically significant demographic characteristics and the racial/ethnic variables. This was done by multiplying each racial/ethnic measure by the other characteristic (e.g., the category Asian American by the category high school graduate to yield a variable Asian/high school). Model 3 results should be the most realistic and interesting.

## Results

### Sample characteristics.

A total of 1,511 of the 1,513 respondents indicated their race/ethnicity: 28.5% white (*n* = 431), 25.7% black (*n* = 388), 19.3% Asian (*n* = 292), and 26.4% Hispanic (*n* = 400). The sample as a whole was not expected to represent the state’s population as a whole, and it did not because the sampling was biased toward nonwhite populations. The sample was designed to be representative of the four major racial/ethnic subpopulations. Using the summary file 4 of the U.S. 2000 census, I observed only small differences between the sample and the four racial/ethnic populations with respect to education, income, age structure, and respondent sex. That is, non-Hispanic whites in the sample closely resembled their New Jersey non-Hispanic white counterparts, non-Hispanic blacks closely resembled the non-Hispanic black population of New Jersey, and so on (U.S. Bureau of the Census 2000).

I compared the results produced by the four sets of samples with the sample data weighted by the four sets of statewide racial/ethnic characteristics for the year 2000. For example, unweighted non-Hispanic black results for the environmental concern scale were compared with weighted non-Hispanic black results. The biggest difference was 6.4% in the direction of the higher concern among unweighted black respondents compared with the weighted black respondent results. Yet, given the 5-year time difference between the census and the sample data and the reality that the biases introduced by the sample are not marked, I present only the unweighted results.

### Question 1: a comparison of environmental concern among four racial/ethnic groups.

Table 1 compares the concern of respondents by race/ethnicity. The results are presented for each of the five aggregate concern measures and the 21 individual components of environmental concern: *a*) problems, *b*) call for action, *c*) government trust, *d*) personal support, and *e*) concern about other problems. Looking first at the environmental problems aggregate of seven indicators, blacks and whites had the highest concern for all seven, and overall a higher problem score. The results of the summary question (“overall”) is telling. More than 40% of whites and blacks classified New Jersey’s environmental problems as “very serious” compared with 25 and 19% for Hispanic and Asian Americans, respectively.

With regard to the call for action questions, whites and blacks again had an overall aggregate score significantly higher than those of their Hispanic and Asian counterparts. The results, however, were inconsistent. Black respondents had the highest proportion of concern that the federal (77%), state (63%), and local (63%) governments were not doing enough to protect the environment. Yet, along with Hispanics (54%), black respondents (58%) had a relatively low proportion of those who were willing to sacrifice economic growth to maintain environmental regulations. These results suggest socioeconomic status confounding because, as a whole, blacks and Hispanics are poorer than their white and Asian counterparts.

With regard to being an active supporter of the environmental movement, there was no measurable difference among the four groups. However, what was notable about this self-declared measure was that the largest proportion of self-identified “active supporters” was among blacks (31%) and Hispanics (29%). By comparison, only 23% of non-Hispanic whites claimed to be “active supporters,” representing quite a difference from the 1970s when whites disproportionately comprised the core environmental support group.

The three trust and control indicators showed virtually no difference between the racial/ethnic groups with regard to personal control over health risks. But with regard to trust in the New Jersey State Department of Environmental Protection, blacks clearly were the least trusting, whereas whites were the least trusting of science to settle differences of opinion about risks. Again, this was an interesting difference worthy of follow-up.

The last comparison was among “other concerns.” Hispanics and blacks had significantly higher concern than did their white and Asian counterparts. The first two were notably more concerned than the last two about rats, obesity, urban redevelopment, and drug-related crime. Whites were the most concerned only about traffic congestion. Asian-American respondents did not have the highest concern for any of the six nonenvironmental concerns. These observations imply geographic residential differences because blacks and Hispanics disproportionately live in New Jersey’s dense urban centers where rats, crime, and urban redevelopment are more likely to be immediate concerns. Traffic congestion, on the other hand, in New Jersey is most often an issue among suburban dweller commuters, who disproportionately are whites.

It is fascinating that African Americans come closest to fitting the 1970s profile of environmental concern described above: call for action, concern about environmental problems, lack of trust of officials to protect the environment, concern about other societal issues, and highest proportion of self-declared active supporters of environmental protection. Asian Americans were the least concerned group. That is, they were least concerned about problems, least interested in government action, least likely to be active supporters, more trusting of government officials, and least concerned about other issues. The fact that black respondents were the strongest supporters of environmental protection is not surprising given the leadership of the Congressional Black Caucus and individuals such as Bob Bullard and Paul Mohai at the national and international levels (Bullard 1994, 2002; Bryant and Mohai 1992).

Whites were similar to their black counterparts in their concerns, with one exception: They were less concerned about the set of other problems (crime rates, urban redevelopment) than were black respondents. Hispanics were similar to Asians in their concerns, with two exceptions: They were more concerned about the environment and much more concerned about other issues than were their Asian counterparts.

### Question 2: demographic correlates.

Full entry and stepwise regression analyses were computed. To conserve space, I present the stepwise results only for model 3. Model 1 assumes that only race/ethnicity is known. The model essentially confirms the results of Table 1—that is, that black and white respondents were more concerned than were Asians and Hispanics about the environment. The multiple *r*-value for the model was 0.242, indicating that other factors need to be included.

Model 2 assumes that we do not know race/ethnicity but that we do know other demographic characteristics. The strongest correlate was rating of New Jersey as a place to live. Those most concerned tended to live in the state for at least two decades and were not elderly. The concerned respondents tended to be high school graduates and rated their neighborhood as a poor- or fair-quality place to live. Last, the most concerned tend to be self-declared liberals and female and to have responded to the survey in English. These 11 indicators produced a multiple *r*-value of 0.336.

These two ordinary least-squares analyses ignored the reality that race/ethnicity and demographic characteristics interact. Model 3 was by far the most interesting (Table 2). The results can be compared with the demographic patterns of environmental concern identified during the 1970s. Eight of 15 statistically significant correlates in Table 2 were for non-Hispanic whites and blacks. Using these eight and others not selected in the stepwise model, the analysis identified more concerned whites and blacks as perceiving New Jersey as a poor or fair place to live. These respondents were disproportionately self-declared political liberals, not political conservatives, more formally educated, < 65 years of age, and female. These results show that the demographic predictors of environmental concern observed during the 1970s exist more than three decades later, with one exception. White women were more concerned than were their male counterparts, but this was not the case among blacks.

With regard to respondents, the 1970s demographic profile that fits whites and blacks did not fit Asian-American respondents. Those who were more environmentally concerned were not disproportionately female, nor were they more formally educated, have higher income, or disproportionately self-identify as liberal. With regard to Hispanics, model 3 shows that the most concerned felt that the state environment and their neighborhood were not good quality, and they disproportionately were politically liberal. Without doubt, the most interesting observation was that those who took the survey in Spanish were less concerned.

Finding that Asians and Hispanics who answered the survey in a language other than English were less concerned about the environment prompted additional analyses. Further testing could not be done for Asian respondents because only 16 of the 292 responded in a non-English language. Forty percent of Hispanic respondents (162 of 400) answered the survey in Spanish, and the remainder in English, and so these data were reanalyzed. The results showed that those who answered the survey in Spanish had much lower environmental pollution concern than did their counterparts who answered the questions in English. For example, the Spanish-language response group had a total problem score of 1.99 (out of 7) compared with 3.26 for those who answered the questions in English. They had a call for environmental action score of only 1.60 (of 4) compared with 2.34 for their counterparts. In the context of the entire survey, those Latinos who took the survey in English were almost identical to their white counterparts (3.31 vs. 3.26; 2.40 vs. 2.34). The 162 who took the survey in Spanish, on the other hand, had much lower environmental pollution concerns than any other subpopulation.

These findings were further traced back to two groups that were notably different. The Spanish-language respondents were older (average age of 45 years), only 47% had graduated from high school and 11% college, and 80% had household income < $35,000 in 2004. In contrast, the average English-speaking Hispanic respondent was 37 years old, 83% had graduated from high school and 43% college, and only 31% had a household income ≤ $35,000. Despite the fact that the Spanish-language response group was on average 9 years older, < 1% had lived in the United States for their entire life and half had lived in New Jersey for < 10 years. In contrast, 37% of their English-primary counterparts had lived in the state for their entire life and only 26% for < 10 years. Finally, their sources of information about the environment are different in interesting ways. The Spanish-language response group relied heavily on television (84%), radio (44%), and family and friends (39%). Newspapers ranked fourth, with 38%. Their English-speaking Hispanic counterparts, like their English-primary counterparts who were European, African, and Asian, about equally relied on newspapers and television (both > 50%), with radio third and family and friends a distant fourth (28%). These data suggest an acculturation explanation; that is, the Spanish-language respondents have not adopted the U.S. mainstream view of environmental protection.

Before accepting this possibility, socioeconomic status and location explanations were pursued and rejected as plausible alternative explanations for these findings. The difference between English- and Spanish-language Hispanics with regard to environmental concern persisted among relatively poor and more affluent subpopulations, among more and less educated, and among residents of New Jersey’s larger cities and other environments. With regard to environmental concern about pollution, the English-language Hispanic respondent subpopulation was much more like their white counterparts than they were like their poorer, less formally educated, and older Spanish-language counterparts who were less concerned about environmental pollution.

## Discussion

There is no question that the racial and ethnic characteristics of the United States population are changing. In 2000, black, Native, Asian, and Hispanic Americans were 28% of the total (78 of 275 million), and they were probably undercounted. The U.S. Bureau of the Census prepared three sets of population projections (Day 1996). The middle set projects the national population to reach 394 million by the year 2050 (Day 1996). The non-Hispanic white proportion is expected to grow only about 10 million and actually decline after 2030. The Census Bureau projects that it will constitute 53% of the national population in the year 2050, compared with 72% in 2000. In contrast, the nonwhite population is expected to increase by > 100 million and reach 47% of the population by 2050. Nearly all the growth is expected to be Hispanics (from 11 to 24%) and Asians (from 4 to 8%). Also, Asian and Hispanic Americans will constitute the bulk of the nonelderly population and become major contributors to the national tax base (Day 1996).

The sheer magnitude of the expected demographic change calls for consideration of the implications of any obvious differences in environmental concern among these two rapidly growing populations and the mainstream. This study shows that white, black, and English-language Hispanics have become anchors of public concern about the environment. Even during a period when public attention has been focused on war, terrorism, and the economy, these populations, especially their educated and liberal elements, are as concerned about environmental protection as their counterparts of the 1970s. Recapitulating, 44% of blacks, 41% of whites, and 35% of Hispanic English-language respondents considered environmental problems in New Jersey to be “very serious.” The comparable numbers for Spanish-language Hispanics and Asian respondents were only 11 and 19%, respectively.

Before considering the policy and actions that are suggested by these observations, I review three key limitations of the study. In light of the results, it would have been useful to ask nationality and a set of questions that measure acculturation. I hope to fill this void in the near future by conducting surveys of Asian (Indian, Chinese, Korean, Japanese) and Hispanic Americans (Cuban, Mexican, Puerto Rican). Second, our “environmental” questions with one exception all relate to pollution. Arguably, if we had asked respondents about their concerns about fishing, hunting, and other forms of recreation and building and cleaning of parks, we would have gotten different answers. In this regard, we have just completed an analysis of a data set that includes these kinds of outdoor ecologic questions, and we have initially found that Spanish-language Hispanics are the most concerned about these. Mental models merge together attitudes, beliefs, experiences, trust, knowledge, impressions, and images (Eagly and Chaiken 1993; Slovic et al. 1986). In short, future surveys need to include environmental pollution and ecologic questions to capture culturally dependent mental models.

The third limitation is that the study area was New Jersey. New Jersey’s environmental protection programs have been among the strongest for decades, reflecting a legacy of industrialization and high cancer mortality (Conservation Foundation 1984). This past makes New Jersey a place where I would expect a great deal of concern about environmental protection. Studies need to be done in other states with many Hispanic and Asian Americans, such as California, Florida, Illinois, and Texas.

Acknowledging these limitations, I return to the question posed in the title of this article: How much difference do race and ethnicity make? The answer depends on where you fit on an optimism–pessimism scale. As we approach the 35th anniversary of the first Earth Day, a solid core of Americans in New Jersey, the most affluent state, are anxious about the future of the nation’s environment. Support is no longer obviously much higher in European Americans than in other groups, and the fact that black and English-speaking Hispanics were equally concerned is good news because it means that environmental concern is not just a special interest issue. Third, an optimist would assume that the current level of support across the entire population is at a low point in the environmental concern cycle, and that environmental pollution concern will increase when the economy is stronger and the nation is less focused on terrorism and war. Finally, even if two subpopulations are less supportive than their counterparts, currently Spanish-language Hispanics and Asian Americans comprise only about 1 of 10 Americans, and they will not reach 20% of the national population until 2025–2030. Hence, an optimist would say that there is time to address the gap between their level of concern and the mainstream’s; that is, they will gradually become mainstream Americans with regard to attitudes toward environmental protection.

A pessimist would find too many hopeful assumptions in the optimist’s assertions. The current low point in concern about pollution could continue because of terrorism and war, the economy, and other domestic issues such as health care costs and energy. Arguably, the 1980s and 1990s were an unusually supportive context for environment management concerns, and that context will not be replicated in the near future.

We can at least take two small steps. First, we need to present a message that does not pit economic health against environmental protection. Globalization has led to the closing of hundreds of thousands of manufacturing facilities in the United States, and services have replaced manufacturing as the primary source of jobs in a increasingly information-dependent and competitive international economy. In an economy in which we are heading to < 10% manufacturing jobs, we need to send a message that a clean environment is a prerequisite for economic competitiveness: Unless you support environmental protection, you will not get high-quality jobs and the services that follow them. Second, we must try to understand better what elements of the environment are of greatest concern to each American subpopulation. This means surveys such as this one, focus groups, and dialogues with leaders that go far deeper than the groups analyzed here. We need to understand what environmental health issues resonate with Mexican, Cuban, Puerto Rican, Dominican, Pakistani, Korean, Chinese, and other Hispanic-American and Asian-American populations. It is my hope that this commentary has underscored the need for such targeted studies.

## Figures and Tables

**Table 1:** Environmental concern by race/ethnicity (mean or mean ± SD).

Concern	Non-Hispanic white (*n* = 431)	Non-Hispanic black (*n* = 388)	Asian American (*n* = 292)	Hispanic American (*n* = 400)	One-way ANOVA *F*-value
Overall environmental concern					
Total score (minimum 5, maximum 30)	17.57 ± 4.75	18.32 ± 4.64	15.02 ± 4.41	16.72 ± 4.55	32.4*
Average factor score	0.09 ± 1.01	0.28 ± 0.96	−0.44 ± 0.94	−0.07 ± 0.95	32.2*
Concern (0 = least, 7 = most)	3.31 ± 2.30	3.27 ± 2.13	2.03 ± 1.88	2.74 ± 2.21	27.0*
(1 = very concerned, 0 = not very concerned)					
Overall (0 = no, 1 = yes)	0.41	0.44	0.19	0.25	
Toxic waste (0 = no, 1 = yes)	0.58	0.53	0.34	0.47	
Water pollution (0 = no, 1 = yes)	0.47	0.49	0.31	0.40	
Open space (0 = no, 1 = yes)	0.57	0.41	0.38	0.38	
Garbage (0 = no, 1 = yes)	0.47	0.47	0.28	0.39	
Beach pollution (0 = no, 1 = yes)	0.33	0.40	0.21	0.38	
Air pollution (0 = no, 1 = yes)	0.48	0.54	0.32	0.47	
Call for action (0 = no call, 4 = all actions)	2.40 ± 1.20	2.61 ± 1.27	2.03 ± 1.27	2.04 ± 1.33	18.5*
Maintain regulations (0 = no, 1 = yes)	0.73	0.58	0.69	0.54	
Federal (0 = no, 1 = yes)	0.71	0.77	0.60	0.62	
State (0 = no, 1 = yes)	0.55	0.63	0.40	0.45	
Local (0 = no, 1 = yes)	0.41	0.63	0.35	0.43	
Supporter (1 = active supporter, 4 = active opponent)	2.98 ± 0.80	3.11 ± 0.77	3.04 ± 0.68	3.01 ± 0.84	2.0
Trust (3 = most, 12 = least trusting)	6.41 ± 1.80	6.32 ± 2.00	5.89 ± 1.67	5.88 ± 1.98	9.3*
Trust state (1–4)	2.06	2.42	2.13	2.03	
Trust science (1–4)	2.53	2.01	1.89	1.98	
Control (1–4)	1.82	1.89	1.86	1.87	
Other concerns (0 = none, 6 = all)	2.47 ± 1.52	3.01 ± 1.57	2.03 ± 1.61	3.05 ± 1.78	29.3*
Traffic congestion (0 = no, 1 = yes)	0.71	0.53	0.58	0.53	
Rats and other vermin (0 = no, 1 = yes)	0.14	0.39	0.17	0.39	
Terrorism (0 = no, 1 = yes)	0.42	0.43	0.30	0.57	
Obesity (0 = no, 1 = yes)	0.47	0.53	0.37	0.59	
Urban redevelopment (0 = no, 1 = yes)	0.26	0.40	0.23	0.37	
Drug-related crime (0 = no, 1 = yes)	0.46	0.73	0.38	0.61	

* One-way ANOVA *F*-value significantly different at *p* < 0.05.

**Table 2:** Ordinary least-squares regression of environmental concerns: more complex model 3.

Race/ethnicity and other characteristics	B ± SE	β-Value
Constant	−0.502 ± 0.053	—
Non-Hispanic black respondent and New Jersey as a place to live (1 = excellent, 4 = poor)	0.241 ± 0.041	0.264
Non-Hispanic white respondent and New Jersey as a place to live (1 = excellent, 4 = poor)	0.237 ± 0.043	0.247
Hispanic respondent and New Jersey as a place to live (1 = excellent, 4 = poor)	0.149 ± 0.058	0.153
Non-Hispanic white respondent and self-declares conservative political view	−0.457 ± 0.108	−0.113
Hispanic respondent and survey in non-English language	−0.421 ± 0.094	−0.130
Asian respondent and survey in non-English language	−0.715 ± 0.229	−0.075
Asian respondent and resident for ≥ 20 years	0.556 ± 0.124	0.118
Non-Hispanic black respondent and respondent is ≥ 65 years of age	−0.370 ± 0.129	−0.071
Asian respondent and respondent is ≥ 65 years of age	−0.633 ± 0.241	−0.065
Non-Hispanic black respondent and high school graduate	0.322 ± 0.106	0.133
Non-Hispanic white respondent and self-declares liberal political view	0.239 ± 0.113	0.057
Non-Hispanic white respondent and respondent is ≥ 65 years of age	−0.326 ± 0.108	−0.079
Non-Hispanic white respondent and respondent is female	0.162 ± 0.066	0.117
Hispanic respondent and self-declares liberal political view	0.243 ± 0.108	0.058
Hispanic respondent and rating of their neighborhood as a place to live (1 = excellent, 4 = poor)	0.110 ± 0.055	0.117

These are stepwise analyses in which the *p*-value for inclusion was ≤ 0.05. The multiple *r*-value for model 3 was 0.400; adjusted *r*^2^-value was 0.151. The B-values are unstandardized regression coefficients that are not directly comparable, whereas the β-values are standardized so that they are comparable.
